# Preparation and Characterization of Non-Crimping Laminated Textile Composites Reinforced with Electrospun Nanofibers

**DOI:** 10.3390/nano13131949

**Published:** 2023-06-27

**Authors:** Jaymin Vrajlal Sanchaniya, Inga Lasenko, Sai Pavan Kanukuntla, Anunand Mannodi, Arta Viluma-Gudmona, Valters Gobins

**Affiliations:** 1 Mechanics and Biotextile Research Laboratory, Riga Technical University, 3/3-20 Pulka Street, LV-1007 Riga, Latvia; 2 Department of Theoretical Mechanics and Strength of Materials, Institute of Mechanics and Mechanical Engineering, Riga Technical University, 6B Kipsala Street, LV-1048 Riga, Latvia; 3 Laboratory of Environmental Genetics, Institute of Biology, Faculty of Biology, Latvian University, Jelgavas Street 1, LV-1004 Riga, Latvia

**Keywords:** nanofiber-laminated composite fabrics, electrospinning, non-crimping fabrics, textiles

## Abstract

This research investigated the use of electrospun nanofibers as reinforcing laminates in textiles to enhance their mechanical properties for use as smart and technical textile applications. Crimping plays a crucial role in textiles. Because of crimp, fabrics have extensibility, compressibility, and improved quality. Although crimping is inevitable for fabrics used in smart textiles, it is also a disadvantage as it could weaken the fibers and reduce their strength and efficiency. The study focused on preparing laminated textile composites by electrospinning a polyacrylonitrile (PAN) polymer onto textile fabric. The research examined the effect of electrospun nanofibers on the fabric by using a tensile testing machine and scanning electron microscopy. The results revealed that the prepared laminated textile was crimp-free because of the orientation of the nanofibers directly electrospun on the fabric, which exhibited perfect bonding between the laminates. Additionally, the nanofiber-reinforced composite fabrics demonstrated a 75.5% increase in the elastic moduli and a 20% increase in elongation at breaking. The study concluded that the use of electrospun nanofibers as laminates in textile composites could enhance the elastic properties, and prepared laminated composites will have the advantages of nanofibers, such as crimp-free elastic regions. Furthermore, the mechanical properties of the laminated textile composite were compared with those of the micromechanical models, providing a deeper understanding of the behavior of these laminated composites.

## 1. Introduction

In recent years, there has been an increase in the demand for textiles that can serve as human-adaptive smart (electronic) skins [1], especially for applications involving flexible wearable electronic sensors and devices. These devices, which include wearable health monitors [2,3], health diagnostic instruments, and multifunctional robot skins [4], must be able to mimic the properties of human skin. This involves converting these stimuli into electrical signals and monitoring various physical parameters such as pressure, strain, flexion, movement, deformation, the distribution of spatial pressure, and even contactless sensing, such as the proximity of a finger [5,6].

However, traditional fabrics have disadvantages when used for such applications. Due to the wavy (3D) structure of the fabric, the crimping effect experienced by these fabrics reduces their efficiency (physical and mechanical parameters) [7]. This restriction applies not only to the health industry but also to smart textiles in which fabric yarns are reinforced with nanoparticles [8,9,10]. When stretched, these materials lose a substantial amount of their efficiency.

Nanofibers have recently emerged as a promising material for medical applications [11], such as wound dressings [12,13], drug delivery [14], and tissue engineering in scaffolds [15,16]. Nanofibers have several advantageous characteristics, such as a high surface-to-volume ratio, lightweight, and flexibility. However, one disadvantage of utilizing nanofiber mats is their typically low strength when the nanofibers are applied in a random orientation. In nanofiber-laminated composite materials and fiber-reinforced composite materials, the orientation, length, and diameter of the fibers play a vital role in the elastic response of the composite materials [17,18,19]. 

Considering the benefits of both PAN nanofibers [9,20,21,22] and textiles, the authors of this study sought to develop nanofiber-laminated textile composites. Prepared nanofiber-laminated textile composites could be used for enhanced smart, functional, and antimicrobial textiles. It was expected that these composites would surpass the limitations of conventional fabrics by maintaining their elastic properties in the crimped region under low rates of strain, similar to those of nanofibers, while also exhibiting greater durability than nanofiber mats alone. The successful development of nanofiber-laminated textile composites could lead to advances in human-adaptive smart (electronic) skins, allowing for more efficient and reliable monitoring of various physical parameters while maintaining the desired flexibility and durability.

Most research on nanofibers reinforced with textiles has focused on different properties of the prepared composites; only limited studies have observed the changes in the mechanical properties of the prepared composites. For example, the work most similar to this present study, which was conducted by Jalalah et al., prepared nanofiber/textile composites using PA6 nanofibers fabricated by a needleless electrospinning process, and the change in the behavior of the stress–strain curve of the composite in the elastic region was unnoticed. The reason why this change remained unnoticed could have been that the aggregated nanofibers were not oriented in a single direction [23]. Xiaolu et al. prepared a stretchable electronic capacitive fabric skin by weaving yarn with a coating of electrospun nanofibers, which showed the advantages of using nanofiber-coated yarns for sensing 0.001 N in a short period of time (<50 ms). This recently developed electronic fabric was also suitable for voice recognition and non-contact monitoring airflow, and no changes in the crimp region of the composite fabric were noticed [1]. Jin et al. prepared durable skin-tight electronic textiles using polyvinylidene fluoride (PVDF) nanofibers and evaluated the cyclic stability of the composite material [2]. Another study carried out by Guan et al. prepared structured, breathable, washable, and wearable woven triboelectric nanogenerators utilizing electrospun nanofibers for harvesting biomechanical energy and self-powered sensing [24]. Qiu et al. showed that using nanofibers embedded in textiles could produce durable antibacterial properties [8]. Chen et al. prepared the laminated composite fabric known as URETEK3216LV, but the results revealed that the prepared composites had a crimp that was the same as that of plain fabric, and no changes were observed in the crimped region [25]. Kucukalo-Ozturk et al. prepared a similar nanofiber-laminated composite fabric where nanofibers were collected on a stationary plate, and the composite thus developed was used for acoustic applications [26]. 

Other research groups have developed methods for preparing crimp-free fabric, but no methods have considered incorporating nanofibers in the fabrication of composites. Hahn et al. reviewed methods for the development of stitch-free and non-crimping textiles that did not include the advantage of nanofibers [27]. Bhudolia et al. showed the effect of stitches on the response of non-crimping composite fabrics [28]. 

The authors’ previous experience [29] with textiles laminated with direct electrospun PA6 nanofibers showed that there is no significant adhesion between the textile and the electrospun nanofiber mat. At the ultimate tensile strength after breaking, the nanofiber mats remained elongated and delaminated from the textile. This led to the problem of adhesion in the interface between the textile and the nanofiber mat. To enhance the adhesion between the electrospun nanofiber mat and the textile, solvent-free textile glue was used, which also did not affect the nanofiber mat or the textile fabric. 

To summarize, this article presents a novel method for creating crimp-free textiles using direct electrospun nanofibers on the textiles and an examination of their mechanical properties. The results of this study could potentially pave the way for the development of new high-performance materials that are lightweight and strong. By investigating the properties of these materials, we hope to contribute to the advancement of textile technology and inspire further research in this field. 

## 2. Materials and Methods 

### 2.1. Materials 

In this research, to prepare laminated textile composites reinforced with electrospun PAN nanofibers, electrospun nanofibers were produced using polyacrylonitrile (PAN) powder and N,N–dimethylformamide. Polyacrylonitrile (typical average MW, 150,000; CAS number 25014-41-9), N,N–dimethylformamide (DMF), and ACS reagent (solvent; ≥99.8%; CAS number 68-12-2) were obtained from Sigma-Aldrich (Merck KGaA, Darmstadt 64287, Germany). The fabric (model T561; weave: plain interlacing, linen warp of 28 Tex, cotton weft of 20 Tex + amber fibers of 7.8 Tex; density, 115 ± 6 g/m^2^) was ordered from AB Linas (S.Kerbedzio str.23, LT-35114 Panevezys, Lithuania). Solvent-free fabric glue (Art. 639820) was purchased from Gutermann GmbH (Landstr. 1, DE–79261 Gutach-Besigau, Germany).

### 2.2. Fabrication of Nanofiber-Laminated Composite Fabrics

**Figure 1:**
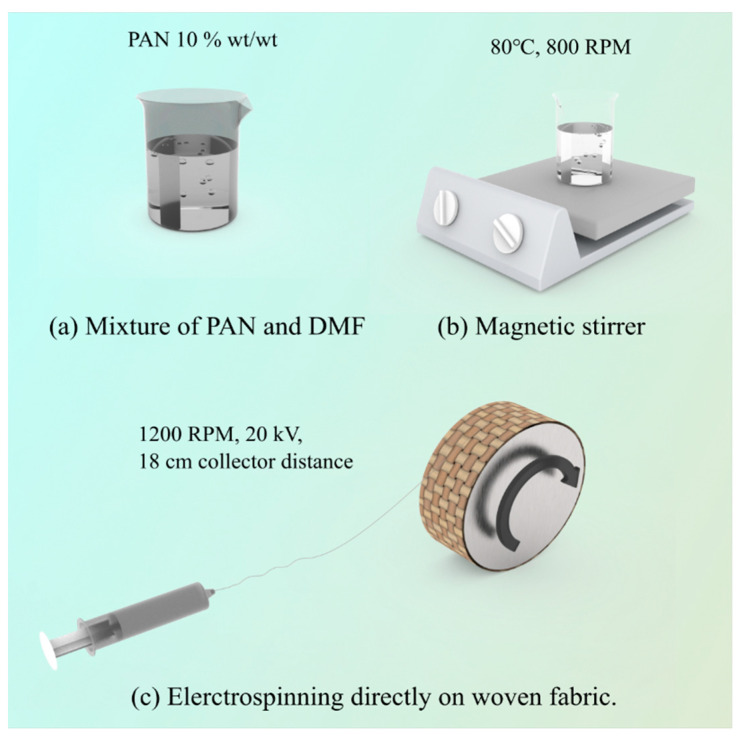
The fabrication process of PAN nanofiber-laminated composite fabrics: (**a**) mixture of PAN and DMF; (**b**) magnetic stirrer; (**c**) electrospinning directly on woven fabric.

The PAN solution was prepared by adding the PAN powder to the solvent (DMF) at 10% *w*/*w* and mixing it for 8 h with a magnetic stirrer (Thermo Scientific™ Cimarec+™ Stirring Hotplates Series, Waltham, MA, USA) at +80 ± 3 °C (room temperature was +22 ± 1 °C; humidity was 60%) and a stirring speed of 800 rpm (Figure 1a,b). It was left at room temperature for 1 h to remove air bubbles and stabilize the solution.

To prepare the laminated textile compound, the fabric was precisely cut to the same length as the periphery of the rotating drum (45 cm × 5 cm), with a longer dimension in the direction of the weft. This fabric was covered on a rotating drum collector (RC-5000; diameter, 140 mm; length, 50 mm; Shenzhen Tongli Tech Co, Ltd., D-608, Shenzhen, China). Fabric glue was applied to the fabric (~208 g/m^2^) via the manual lay-up method. It was measured by weighing the mass of the glue before and after application to the fabric. To test the PAN nanofiber mat, separately, a 5 cm × 5 cm square of aluminum foil (thickness 35 µm; Vireo.de, Merseburg 06217, Germany) was kept on the glue; therefore, nanofibers could be collected later, and any effect of the glue on nanofibers could be avoided.

The PAN nanofibers were spun (Figure 1c) at a room temperature (+22 ± 1 °C) using an electrospinning setup: a Fisherbrand™ single-syringe pump, a needle-based electrospinning machine (Danbury, CT 06811, USA), and a pre-prepared rotating drum covered with the fabric. A 10 mL plastic syringe (lure lock) and needle (Type 18 Ga) were used. The electrospinning parameters used in our work were a voltage of 20 kV and a flow rate of 1 mL/h, and the distance between the syringe and the center of the collector drum was 18 cm. The collector drum was constant at 1200 rpm (tangential speed = ~8.8 m/s). For analyses of the mechanical properties, the specimens were collected after 8 h of electrospinning. A scanning electron microscope (Hitachi High-Tech TM Series TM3030 Plus, The Netherlands) was used for the morphological analysis of the samples obtained after 8 h of electrospinning. Before any studies or characterizations were performed, all samples were kept at room temperature (+22 ± 1 °C) and a relative humidity of less than 60% for a period of 48 h (according to ISO 139:1973, Textiles—standard atmospheres for conditioning and testing).

### 2.3. Morphology of the Composite Nanofiber-Laminated Textiles

The scanning electron microscopy (SEM) images were taken with a TM300 tabletop microscope (Hitachi) with a magnification of 1500 mm and a vacuum of 10-2 Torr, and a coating of 6 mA gold (Au) ions with a thickness of 150 Å, as mentioned in [19]. The nanofibers’ orientation was determined using the OrientationJ plug-in in ImageJ software (version 1.53 t) [30,31,32] (ImageJ, National Institutes of Health, Bethesda, MD, USA). The average diameter of the nanofibers and the standard deviation was determined by measuring the diameter of 100 nanofibers randomly selected from three SEM images. The thickness of the nanofiber mat, the laminated textile composite, and the fabric was determined by a digital micrometer (range: 0–25 mm; Digimatic micrometer, MDC-25PX. code No. 293-240-30, serial No. 71912410, Mitutoyo, Japan) with a sensitivity of 0.001 mm and a low measuring force of 5 N.

### 2.4. Tensile Properties

**Figure 2:**
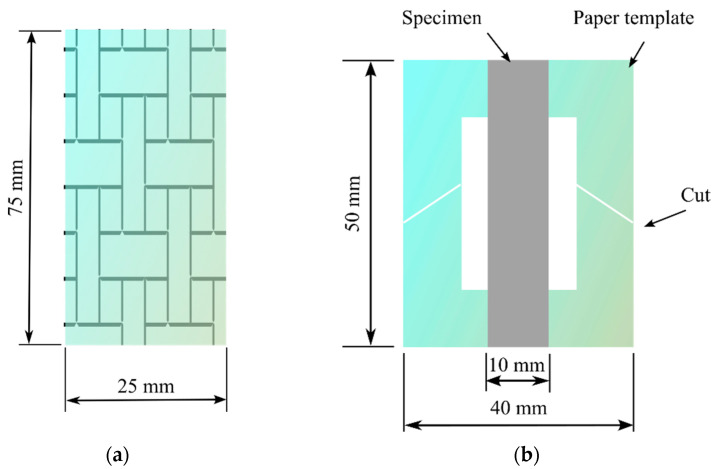
Dimensions of the specimens: (**a**) plain fabric and the composite PAN nanofiber-laminated fabric; (**b**) specimen of the nanofiber mat with a paper template.

The plain fabric and the PAN nanofiber-laminated composites were tested according to the ASTM D2261 standard (strip method), with a specimen size of 75 mm × 25 mm (Figure 2a). A Mecmesin Multi-Test 2.5-i tensile testing machine (PPT Group UK Ltd., t/a Mecmesin, Newton House, Spring Copse Business Park, Slinfold, West Sussex RH13 0SZ, UK) was used with a 2.5 kN sensor to measure the tensile properties. The elongation speed was 50 mm/min, and the testing conditions, according to the ISO 139:1973 standard (Textile—Standard atmosphere for conditioning and testing), were a temperature of +21 ± 1 °C, relative humidity of 60%, and an atmospheric pressure of 760 mm Hg. Five measurements were made to determine the tensile characteristics. The thickness of the test specimens was measured using a digital micrometer. The thickness of the specimen was the mean of the thicknesses measured at three different points. The specimens were cut parallel to the direction of the nanofibers (the weft direction of the fabric). For independent testing of the nanofiber mats, which were collected by interleaving with aluminum foil, the same equipment was used with a 25 N sensor according to the ASTM D882-18 standard at an elongation speed of 5 mm/min. The size of the specimens was 50 × 10 mm (length and width). A similar method to that in [19] was used for testing the nanofiber mat, in which a paper frame measuring 50 mm × 40 mm with an inside cut of 30 mm × 20 mm was created. Both ends of the specimen adhered to the paper frame using double-sided thin Scotch tape (3M Scotch Magic Tape (matte finish), 3/4″ × 36 yards, desk dispenser refill). After attaching the paper frame and specimen to the tensile testing machine, the sides of the paper frame were cut using scissors. Figure 2b shows the specimen with the cut paper frame, which facilitated the mounting of the specimen in the grip of the tensile testing machine and was in line with the usual specimen sizes used for the nanofiber mat.

For statistical significance, the observed tensile Young’s modulus was averaged across at least five specimens. To understand the elastic properties of the prepared nanofiber-laminated composite fabrics [33], we calculated the elastic modulus using micromechanical models. First, the elastic modulus of the nanofiber-laminated fabric was evaluated using the fundamental rule of mixing (ROM) for comparison purposes:

(1) $$E_{C}=E_{FA}V_{FA}+E_{NF}(1-V_{FA})$$

where E_C_ is Young’s modulus of the nanofiber-laminated composite fabric; E_FA_ and E_NF_ are the experimental Young’s modulus for the fabric and PAN nanofiber mat, respectively; and V_FA_ represents the volume fraction of the fabric. The value of E_C_ predicts the linear relationship between the fabric and the nanofiber mat. The volume fraction of the reinforced nanofiber mat was calculated on the basis of the increase in the thickness of the nanofiber-laminated composite fabric; the thickness of the glue was considered to be 50 µm and not considered in the calculation, as it could be varied, depending on the loop.

The tensile modulus was predicted using the Cox–Krenchel micromechanical model. This model, which was based on the classical shear-lag theory, is among the most popular. This model makes the following assumptions: first, the fiber and matrix respond elastically; secondly, there are no axial loads on the fibers’ ends; and lastly, the fiber–matrix interface is ideal. The Cox–Krenchel model is characterized as

(2) $$E_{C}={\eta_{0}E}_{NF}V_{NF}(1-\frac{tanh(ns)}{ns})+E_{FA}(1-V_{NF})E_{FA}$$

(3) $$n=\sqrt{2E_{FA}/\left[E_{NF}(1+V_{FA})ln\frac{1}{V_{NF}}\right]}$$

where E_C_ is the elastic modulus of the nanofiber-laminated composite fabrics; E_NF_ and E_FA_ are the experimental moduli of the PAN nanofiber mat and fabric, respectively, with the fiber orientation factor η_0_ = 3/8, assuming an in-plane isotropic orientation of the fibers in a random short-fiber polymer composite; and *s* is the fiber aspect ratio, where the average length of the fibers L can be used to calculate *s* = L/D, where D is the fibers’ diameter [34].

Similarly, the elastic modulus of the nanofiber-laminated composite fabric was calculated assuming a random in-plane fiber orientation using the Tsai–Pagano model shown in Equation (4) [35]:

(4) $$E_{C}=\frac{3}{8}E_{L}+\frac{5}{8}E_{T}$$

(5) $$E_{L}=E_{NF}V_{NF}+E_{FA}(1-V_{NF})$$

(6) $$E_{T}=\frac{E_{NF}E_{FA}}{E_{NF}\left(1-V_{NF}\right)+E_{NA}V_{FA}}$$

where E_L_ and E_T_ are the longitudinal and transverse modulus of the nanofiber-laminated textile composite, respectively, which were computed longitudinally and transversely in the direction of the fibers, assuming a unidirectional composite with cylindrical fibers.

The Halpin–Tsai model is a mathematical model that predicts the elasticity of composite materials based on the geometry and orientation of the filler, as well as the elastic properties of the filler and matrix. The model is based on the self-consistent field process but is also considered to be empirical. The Halpin–Tsai model is given in Equation (7):

(7) $$E_{C}=\frac{E_{FA}(1+\zeta \eta V_{NF})}{1-\eta V_{NF}}$$

(8) $$\eta =\frac{E_{NF}-E_{FA}}{E_{NF}+\zeta E_{FA}}$$

In Equations (7) and (8), η is a function and ζ denotes an empirical parameter or a curve-fitting parameter which is used to calculate the value that matches the experimental data. In this study, the empirical parameter ζ = 1 was selected, as this is used for a single composite [36,37].

## 3. Results and Discussion

### 3.1. Morphology

The nanofiber-laminated composite fabric produced by electrospinning nanofibers on a rotating drum collector covered with woven fabric exhibited precise lamination and distribution of the nanofibers on the material, as evidenced by their uniform thickness. There were no visible signs of voids or separation between the nanofiber mat and the fabric, which confirmed the good phase morphology. The nanofiber-reinforced fabric was generally flat and straightforward to cut into rectangular specimens for tensile testing.

**Figure 3:**
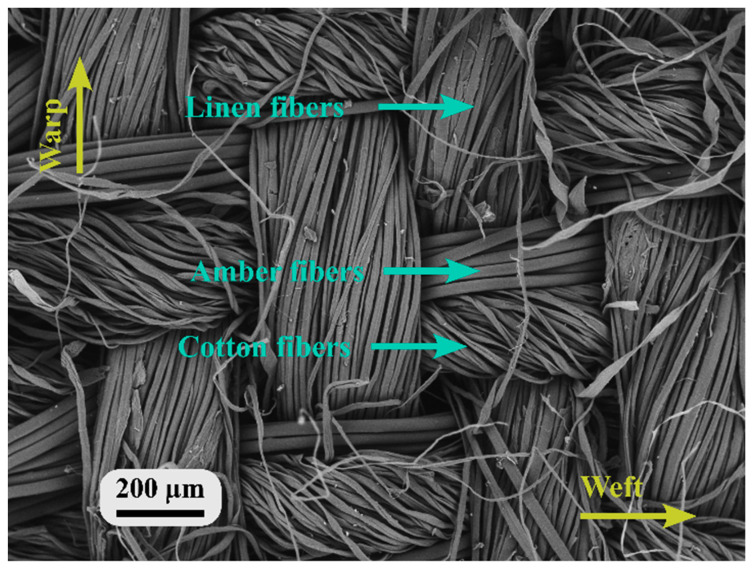
Nanofiber-laminated composite textile (view: outer side).

Figure 3 presents a reinforced SEM image of the fabric-side view of the nanofiber-laminated composite fabric. As can be seen from the SEM image of the weft, two types of yarn are visible, one made of amber fiber and another of cotton fiber. The average diameter of the amber fibers was 25 ± 2 µm, and the diameter of the cotton fibers was 15 ± 1 µm. In the warp direction, which used linen yarns, the filaments had a diameter of 12 ± 1 µm.

**Figure 4:**
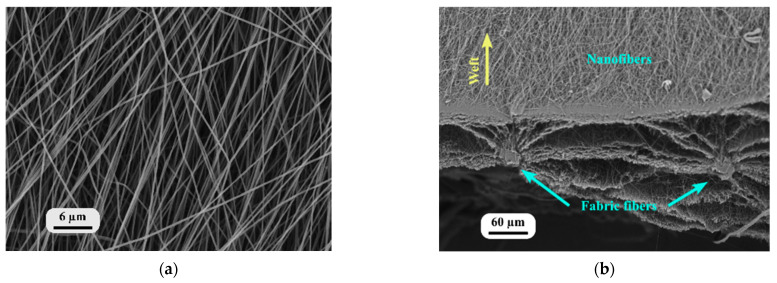
Nanofiber-laminated composite fabrics: (**a**) view from the PAN nanofiber side; (**b**) cross-sectional view.

Figure 4a shows a reinforced SEM image of the nanofiber mat on the fabric from the nanofiber-side view, which was also used to identify the orientation of the nanofibers. There was no droplet formation in the nanofiber mat, which indicated the continuous flow of the PAN solution and the formation of nanofibers from the syringe during electrospinning. Figure 4b presents the edge of the nanofiber-laminated composite fabric, where the intersection of the nanofiber mat and the fabric is visible. The nanofibers created ring-shaped layers (a radial zone between the microfibers) near the fibers of the yarn, which corresponded to the connection between the nanofiber mat and the yarn fibers.

**Figure 5:**
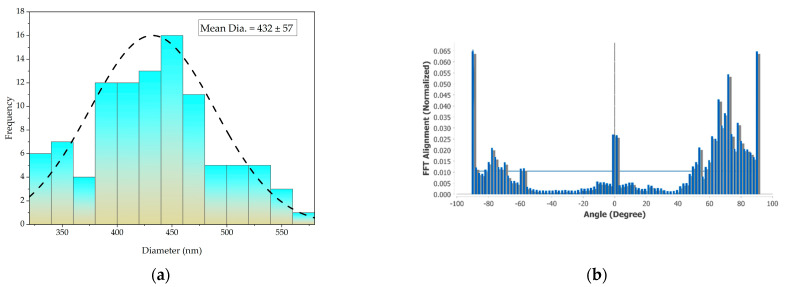
Characteristics of nanofibers in the nanofiber-laminated composite fabric: (**a**) distribution of the nanofibers’ diameter; (**b**) orientation of the PAN nanofibers.

Figure 5a shows graphs of the distribution of the nanofibers’ diameter. The skew of the distribution of the diameters of the nanofibers collected at a constant velocity (|A|) was less than 0.5; hence, a Gaussian distribution could be used. The nanofibers had an average diameter of 432 ± 57 nm. Figure 5b shows the results of an investigation into the alignment of PAN nanofibers laminated onto the fabric. When the drum rotated at 1200 rpm, the investigation revealed that the (normalized) value of the FFT alignment ranged from 0.0 to 0.65. The orientation of the vast majority of the nanofibers was between 0° and 30°. Increasing the drum’s rotational speed at a constant rate stabilized the drawing process and enabled a uniform range of diameter to be obtained [38].

### 3.2. Tensile Tests

**Figure 6:**
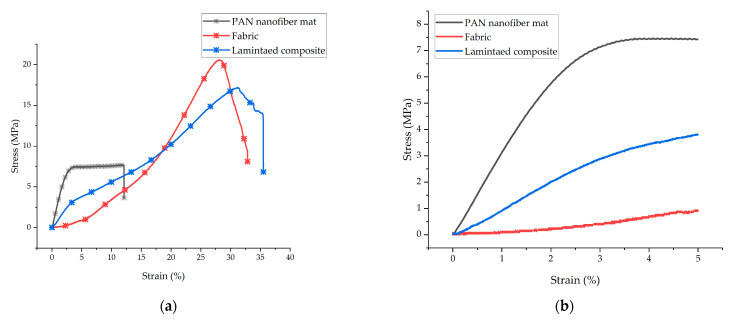
(**a**) Representative stress–strain graph of the nanofiber mat, the fabric, and the laminated composite fabrics; (**b**) enlargement of the stress–strain curve in the low range of deformation (0–5%).

Figure 6a shows the stress (σ) versus strain (ε) graphs of the PAN nanofiber mat, the plain fabric, and the nanofiber-laminated composites. Due to the gripping force, there were no failures of the grip during tensile testing. Figure 6b presents an enlargement of the stress–strain curve in the deformation range of 5%. It is evident from the enlarged graphs of the PAN nanofiber mat, the fabric, and the nanofiber-laminated composite fabric that the elastic moduli of the laminated composite fabric were higher than that of the fabric. It is also evident from the graph that the stress–strain curve of the fabric shows the crimping region where the fabric underwent deformation without carrying a load, but in the prepared nanofiber-laminated composite fabric, the elastic region shows the crimp-free behavior of the nanofiber-laminated composite. Under loading, the fabrics underwent a strain of approximately 10–15% in the crimping region; initially, up to 2.5% strain, no stress was observed (Figure 6b).

The initial thickness of the fabric was 301 ± 12 µm, which increased to 565 ± 13 µm after electrospinning of the fabric. The thickness of the tested PAN nanofibers was 147.8 ± 7 µm, which was less than the extra layer on the fabric after electrospinning, which could be explained by the fabric glue, which had a thickness of 50 ± 2 µm. The nanofibers collected on the interleaved aluminum foil lost some nanofibers as well.

The tensile strength of the PAN nanofiber mat was 10.2 ± 2 MPa. There was no significant change in the tensile strength at breaking in the comparison between the fabric and the nanofiber-laminated composite fabric. For the fabric, the ultimate tensile strength was 18.9 ± 2 MPa; for the nanofiber-laminated composite, it was 17.2 ± 1 MPa. The Young’s modulus of the fabric was 72.9 MPa in the linear region (after crimping in the 10 to 15 MPa range, Figure 6a), while Young’s modulus of the nanofiber-laminated composite was 128 ± 12.5 MPa, which indicated an increase of 75.5%. The nanofiber showed the highest elastic modulus of 333.4 ± 32 MPa. The PAN nanofiber mat underwent 18 ± 4% elongation before breaking. The fabric broke at an elongation of 31.2 ± 2%, whereas the PAN nanofiber-laminated fabric broke at an elongation of 37.5 ± 2%. The nanofibers electrospun onto the fabric achieved an increase of 20% elongation at breaking. The thickness, tensile strength, elastic moduli, and elongation at breaking are summarized in Table 1.

The process of reinforcing the fabric with nanofibers involved electrospinning nanofibers onto the fabric using a high-velocity rotating drum collector. This method promoted the better alignment of the nanofibers, as previously observed by the authors [19]. It is widely known that oriented nanofibers tend to exhibit greater strength compared with randomly collected nanofibers; additionally, nanofibers with a diameter of less than 700 nm provide greater strength. Therefore, we achieved the best strength in the nanofiber-reinforced fabric [39].

When the nanofibers were electrospun on the fabric, perfect adhesion between the fabric fibers and the electrospun nanofibers was achieved (Figure 4b). Because of intermolecular forces and surface interactions, including electrostatic attraction between charged nanofibers and fabric, as well as mechanical interlocking facilitated by the fabric’s physical structure, contribute to the adhesion of nanofibers to the fabric. This perfect adhesion can be attributed to the increased elastic moduli observed in the nanofiber-laminated composite textiles. The adhesion achieved between the fabric and the nanofibers enhanced the transfer of the load between the two materials, leading to an increase in the elastic moduli of the composite.

**Figure 7:**
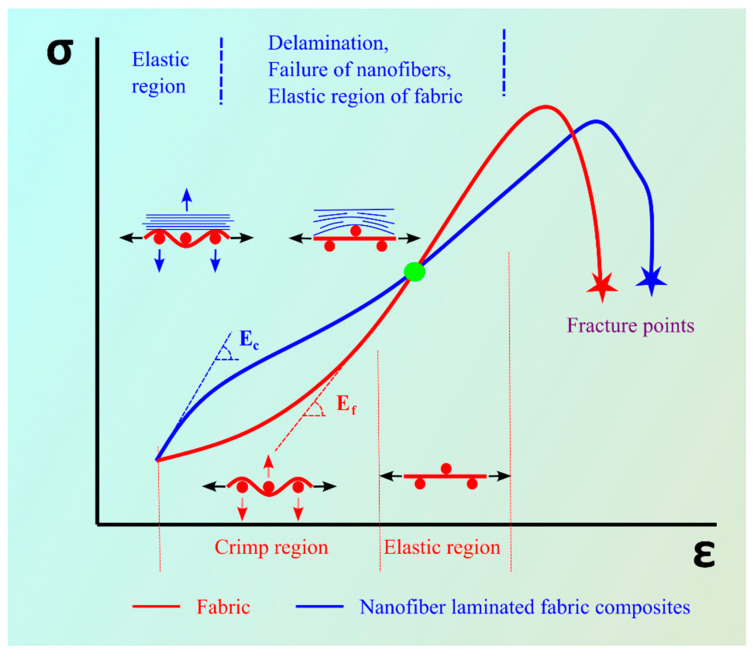
Typical stress–strain curve of the fabric and the nanofiber-laminated composite fabric.

Figure 7 provides a visual representation of the stress–strain behavior of two types of textiles: plain fabric and the nanofiber-laminated composite fabric. This helped us understand the regions of different behavior observed in each material. The red line in Figure 7 represents the stress–strain behavior of the woven fabric. Under loading conditions, the fabric experienced a transition in the crimping region, where the load was not carried effectively. This transition caused an alignment of the woven structure of the yarns. The behavior of the woven fabric can be typically characterized by an elastic region (linear region) followed by a non-linear region.

In the case of the nanofiber-reinforced composite fabric, two distinct behaviors could be observed. The elastic region (the linear region) reflected the initial response of the material to the applied force. However, the nanofiber-reinforced fabric also exhibited a non-linear region due to the delamination and constant elongation of the PAN nanofibers under constant stress. This behavior led to the nanofiber-laminated composite fabric entering the plastic region, where breakage of the nanofibers occurs.

Another region depicted in Figure 7 represents the point (shown in green) where the red line (the woven fabric) and the blue line (the nanofiber-laminated composite fabric) intersect. At this point, it is expected that all nanofibers will be in the plastic region and, due to delamination, the fabric will behave independently, continuing in its own linear region (elastic region).

The observed increase in the percentage of elongation can be explained by the delamination of the nanofiber mat and the fabric. After delamination, some nanofibers may break not only from the center but also from both ends. This behavior is influenced by the woven structure of the fabric, and the nanofibers tend to break at the ends after the failure of the woven loop.

**Figure 8:**
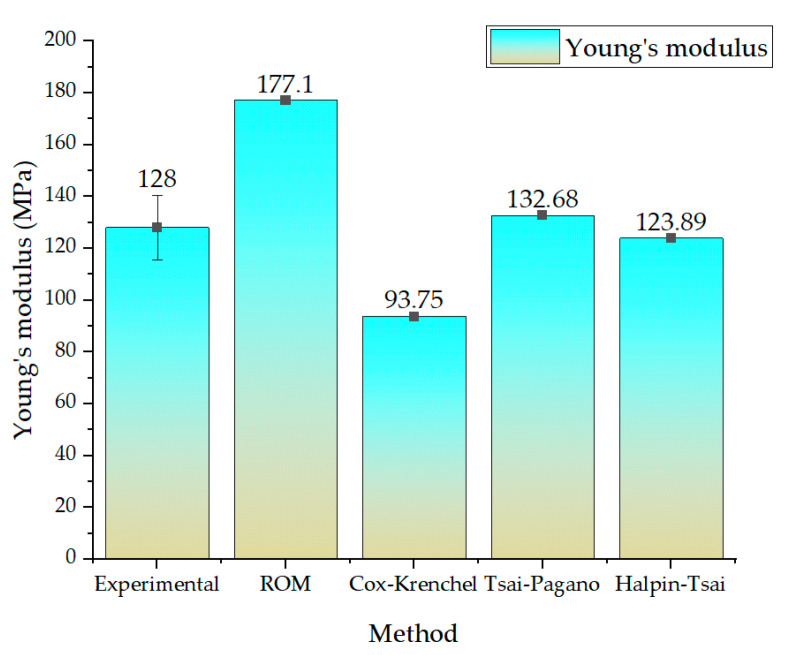
Comparison of micromechanical models and the experimental results.

Figure 8 illustrates the experimentally obtained elastic modulus and a comparison with the theoretical micromechanical model of the nanofiber-laminated composite fabric. The ROM, Cox–Krenchel, Tsai–Pagano, and Halpin–Tsai models were utilized to calculate the theoretical elastic modulus. The ROM model overestimated the elastic modulus of the nanofiber-laminated composite (177.1 MPa), whereas the Cox–Krenchel model underestimated the elastic modulus (93.75 MPa). On the other hand, the Tsai–Pagano micromechanical model and the Halpin–Tsai model made predictions within the range of the experimental results, namely 132.68 MPa and 123.18 MPa, respectively. To determine statistical significance, Student’s t-test was employed. Based on the calculated *p*-values, it is concluded that at a significance level of 0.05, there is no significant difference between the experimental values and any of the predicted numerical values.

The observed overestimation of the elastic moduli by the rule of mixtures (ROM) model can be attributed to the assumption of ROM that considers the average behavior of two independent materials irrespective of their geometry and orientation. In this study, the nanofibers were oriented between 0° and 30° (Figure 5b) and were not completely random, which contradicted the assumption of ROM. Similarly, the Cox–Krenchel model accounted for a random orientation of the fibers, which did not correspond to our experimental setup, and the model underestimated the results. On the contrary, the Tsai–Pagano and Halpin–Tsai models predicted the elastic moduli more closely to the experimental results, with a deviation of 4.68 MPa and 4.11 MPa, respectively, which is within 5% of the experimental results. These models predicted the elastic modulus within a reasonable range, taking the orientation of the composite material into account. A similar pattern was observed in a prior study [40] of micromechanical models of nanofibers, in which the Tsai–Pagano model predicted results within an acceptable range, whereas the ROM overestimated and the Cox–Krenchel model underestimated the result for composites prepared with nanofibers.

The Tsai–Pagano micromechanical model provided a more precise prediction of the elastic modulus within the range of the experimental results. This model accounted for both the random in-plane fibers and the transverse elastic modulus. The Tsai–Pagano model provided a more accurate estimation of the elastic modulus of the nanofiber-laminated composite material by taking these factors into account. Similarly, the Halpin–Tsai model accurately predicted the elastic modulus within acceptable limits. This model takes both the orientation and the geometry of the composite material into account, and the predictions were well within the range of 5% of the experimental results. By incorporating these variables, the Tsai–Pagano and Halpin–Tsai models provide more accurate predictions than the ROM model, which was within the range of 5% of the experimental elastic modulus.

### 3.3. SEM Analysis after Tensile Testing

**Figure 9:**
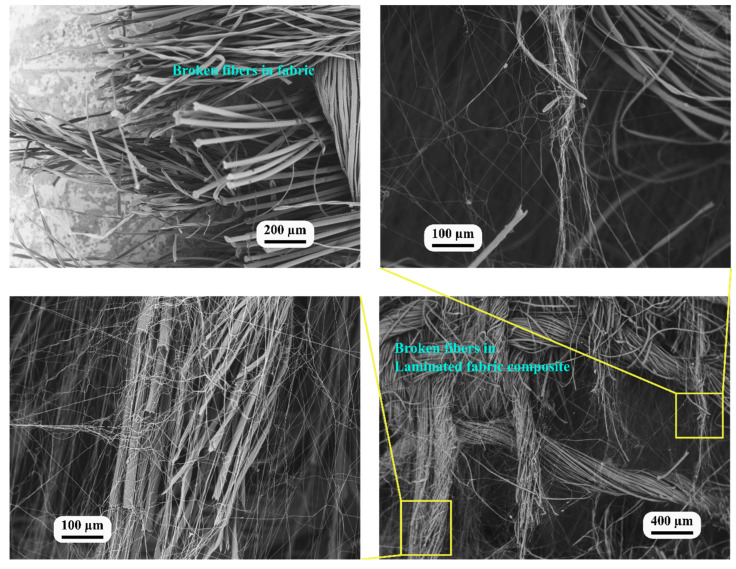
SEM image of test specimens of the fabric and the nanofiber-laminated composite fabric.

SEM was used to examine the morphology of the fractured tensile cross-sections of both the woven fabric and the nanofiber-laminated composite fabric. Figure 9 illustrates the fractured surfaces of these materials, thereby illustrating their fracturing behavior.

The fractured surface of the woven fabric revealed only broken fibers, indicating a mechanism of brittle fracture with deformation occurring within the same plane. In contrast, the fractured surface of the nanofiber-laminated composite fabric revealed the fracturing of individual fibers at different levels and planes. This observation can be explained by several factors. First, it may be due to the fabric glue, which facilitated the direct adhesion of the nanofibers to the microfibers of the yarn, as well as the adhesion of the directly electrospun nanofibers to the microfiber (Figure 4b). As shown in the SEM images in Figure 7, the significant increase in elongation can be attributed to the nanofibers adhering to the microfibers of the yarn. Some nanofibers remained connected to the ends of the microfibers even after fracturing due to the interfacial forces [41].

The SEM analysis provided visual evidence of the nanofibers’ interaction with the microfibers within the yarn’s structure. The presence of connected nanofibers after fracturing indicated that these bonds between the nanofibers and the microfibers remained intact, thereby contributing to the enhanced elongation and mechanical properties of the composite material.

**Figure 10:**
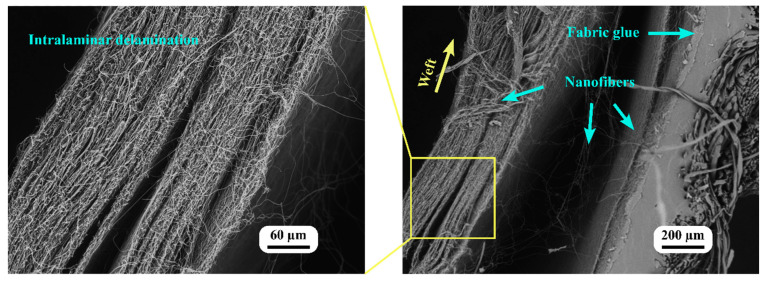
SEM image of a broken specimen of nanofiber-laminated composite fabric (near the grip).

Figure 10 depicts SEM images captured close to the clamps and the end of the warp fibers, which provided valuable insights into the fracture behavior and the presence of shear stress within the nanofiber-laminated composite fabric and the woven fabric.

After fracturing, the SEM images demonstrated that the nanofiber-laminated composite fabric and the woven fabric separated from one another. During elongation, the in-plane shear stress between the layers of the nanofiber-laminated composite fabric caused this separation. The shear stress was generated as the result of mismatched elastic regions between the layers. Due to this shear stress, the laminates separated from one another, resulting in delamination. Even after this separation, some nanofibers remained connected to the adhesive, indicating the strength of the adhesive bond between the nanofibers and the fabric.

Moreover, during elongation, shear stress was observed not only between the layers but also within the nanofiber mat itself. The SEM images demonstrate that the nanofiber mat separated into distinct layers, indicating that the mat itself was an orthotropic material consisting of distinct layers. This separation of the nanofiber mat during elongation further confirmed the presence of shear stress within the microlayers of the nanofiber mat.

It is evident from the images that one of the microlayers remained connected to the fabric glue while the second layer of nanofibers was delaminated. This result indicated that the electrospinning technique used to apply nanofibers directly on the fabric’s surface resulted in sufficient adhesion between the nanofibers and the fabric glue to transfer the load in the crimping region. Even after the delamination of the fabric and the nanofiber mat, the first direct electrospun microlayer of the nanofibers and the adhesive glue maintained a bond, as one microlayer remained attached (Figure 10).

This adhesion between the nanofibers and fabric adhesive was essential for the overall structural integrity and mechanical performance of the nanofiber-laminated composite fabric. Adhesion permits the efficient transfer of the load between the nanofibers and the fabric, enabling them to function as a composite material. In addition, this adhesion contributes to the enhanced mechanical properties observed, such as increased elongation and enhanced elasticity, as discussed in the earlier sections.

## 4. Conclusions

This study examined the use of electrospun nanofibers as reinforcing laminates for smart textiles. The study focused on cost-effective electrospinning polyacrylonitrile (PAN) nanofibers onto a woven fabric to create laminated composite fabrics. The results demonstrated precise lamination and distribution of the nanofibers on the fabric, with no visible voids or separation. As a result of the oriented nanofibers being electrospun directly onto the fabric, the nanofiber-laminated composite fabrics exhibited crimp-free behavior.

The fabric specimen’s (75 mm × 25 mm) mean weight was 274 ± 10 mg, and this value was observed to increase to 571 ± 10 mg after the fabrication of the nanofiber-laminated composite fabric. The results of tensile testing revealed that nanofiber-laminated composite fabric had greater elastic moduli than those of the fabric alone. The nanofiber-reinforced composite fabric demonstrated a 75.5% increase in the elastic moduli and a 20% increase in elongation at breaking. The adhesion between the nanofibers and the fabric’s fibers improved the transfer of the load, resulting in increased elastic moduli.

Among all the micromechanical models considered for estimating the elastic moduli of the nanofiber-laminated textile composite, the Tsai–Pagano and Halpin–Tsai models provided accurate predictions of the elastic modulus compared with the experimental results. On the contrary, both the rule of mixtures (ROM) model and the Cox–Krenchel model misestimated the elastic moduli. This discrepancy highlighted the significance of considering the orientation of nanofibers in laminated composites when predicting their mechanical behavior.

The SEM examination of the fractured surface provided visual evidence of the interaction between the nanofibers and the fabric’s microfibers. Even after fracturing, the presence of connected nanofibers indicated the strength of the adhesive bond, which contributed to the improved mechanical properties. The SEM images also revealed shear stress within the nanofiber-laminated composite fabric and separation of the layers during elongation (this was observed after breaking), confirming the presence of shear stress within the microlayers of the nanofiber mat.

## Figures and Tables

**Table 1:** Tensile properties of the PAN nanofiber mat, the fabric, and the laminated composite.

Materials	Thickness, t (µm)	Ultimate Tensile Strength, σ (MPa)	Young’s Modulus, E (MPa)	Elongation at Breaking, ε (%)
Fabric	301 ± 12	18.9 ± 2	72.9 ± 3	31.2 ± 2
PAN nanofiber mat	147.8 ± 7	10.2 ± 2	333.4 ± 32	18 ± 4
Laminated composite	565 ± 13	17.2 ± 1	128 ± 12.5	37.5 ± 2

## Data Availability

The data presented in this study are available on request from the corresponding author.
